# Willingness of corneal donation and its associated factors among adult patients attending Gondar University Comprehensive and Specialized Hospital

**DOI:** 10.1371/journal.pone.0256102

**Published:** 2021-08-20

**Authors:** Eyerus Gesesse, Sofonias Addis Fekadu, Gizachew Tilahun Belete

**Affiliations:** 1 Department of Optometry, School of Medicine, College of Medicine and Health Sciences, Hawassa University, Hawassa, Ethiopia; 2 Department of Optometry, School of Medicine, College of Medicine and Health Sciences, University of Gondar, Gondar, Ethiopia; Edith Wolfson Medical Center, ISRAEL

## Abstract

**Introduction:**

Corneal transplantation is the only treatment option for corneal blindness to restore sight. However, there is a disproportionate imbalance between the demand and supply of corneal tissue in Ethiopia. This is because accessing corneal tissue is reliant on people who are willing to donate corneas after death.

**Objective:**

This study aimed to assess the proportion of willingness to donate cornea and associated factors among adult patients attending at University of Gondar comprehensive and specialized hospital.

**Method:**

Institutional based cross-sectional study was conducted from July 13 to July 28, 2020, through a face-to-face interview. A total of 451 samples were selected using systematic random sampling. The data were entered into Epidemiological information version 7 and exported to statistical package for social science version 20 for formal analysis. Variables with a P-value of < 0.20 in a bi-variable logistic regression were entered into the multivariable logistic regression and those variables with a p-value of < 0.05 were taken as statistically significant. The strength of association was shown using the odds ratio with a 95% confidence interval.

**Result:**

A total of 408 adults participated in this study with a response rate of 90% and the proportion of willingness to donate cornea was 179(43.90%). Participants who had a religious belief in Christianity (AOR = 3.23 (95% CI: 1.09–9.57)) and good knowledge about corneal donation (AOR = 5.45(95%CI: 2.69–11.18)) were positively associated with the willingness of corneal donation. On the other side, the age group above 43 years (AOR = 0.31(95% CI: 0.11–0.89) was negatively associated with the willingness of corneal donation.

**Conclusion:**

The proportion of willingness to donate cornea 43.9% among participants attending Gondar University Comprehensive and Specialized Hospital. Age group greater than 43 years, religion of Christianity and good knowledge were associated with the willingness of corneal donation.

## Introduction

Visual impairment and blindness are major health-related problems worldwide. They cause a considerable economic burden for the affected person, caregivers, and society at large [1]. It is associated with loss of ability to work, affects the quality of life, and triggers depression [2–4]. Visually impaired individuals were estimated to be 216.6 million in 2015 of which 4.5 million patients experienced moderate-to-severe impairment due to corneal opacity [5]. According to the reports of the world health organization (WHO), there are about 45 million blind people worldwide [6] and corneal opacity constitutes 5% of the total causes of blindness globally [7].

According to a national survey done in Ethiopia (2006), the proportion of blindness and low vision were 1.6% and 3.7% respectively. Corneal blindness accounts for 19.3% of all blind cases in the country and it mainly resulted from trachoma, Xerophthalmia, use of harmful traditional eye medicines, onchocerciasis, and ocular trauma [6, 8].

Corneal blindness affects many people in their most productive years and its impact is also seen in the population at large that leads to a greater total disability-adjusted life year [9]. Fortunately, 80% of visual impairment which is caused by corneal diseases is preventable [10, 11], but once the cornea (transparent front part of the eye) has lost its function(transparency), the best choice to restore vision in the affected eye is by corneal transplantation or grafting [12].

Corneal transplantation has been known as one of the most successful types of tissue grafts [13] and about 100,000 cases of corneal transplants are carried out every year in the world [14]. Furthermore, around 12.7 million people are waiting for corneal transplantation which is 1 in 70 of the needs for grafting are covered worldwide.

In Ethiopia, there is only one eye bank in the country (The Eye Bank of Ethiopia) that is involved in the collection and storage of donor’s cornea, and about 130–150 corneas are harvested every year. This is why only 90–120 transplants are carried out in the country that does not match the needs of recipients. Corneal donation relies on the contest of the individual who is interested or willing to donate. Additionally, he/she will be advised to convince his family about the donation as consent is not taken from the family. Once he/she gives the consent, the trained nurses will pledge the cornea when the individual passed away [15]. All these show a disproportionate imbalance between the demand and supply of corneal tissue and this happens because accessing corneal tissue is reliant on the willingness of people to donate their cornea after death. Lack of awareness and unwillingness to donate cornea results in absence of corneal tissues in eye banks [16]. Different studies indicated that age, gender, educational level, religion, residency, attitude, awareness, knowledge, and ethnicity were found to be significantly associated with the willingness of corneal donation [17–26]. Unwillingness to donate cornea results in greater demand for corneal transplantation which in turn increases the burden of corneal blindness. To overcome this problem individuals should have awareness of corneal transplantation and should be willing to pledge their cornea voluntarily. Knowing the factors affecting willingness to donate cornea could increase the number of available corneal grafts and will give us information on our approach to the community. Therefore; this study aimed to assess the proportion of willingness to corneal donation and factors associated with it among adult patients attending Gondar University Comprehensive and Specialized Hospital.

## Methods and materials

### Study design, setting and period

A hospital-based cross-sectional study was conducted from July 13 to July 28, 2020, at Gondar University Comprehensive and Specialized Hospital in Gondar city. Gondar city is located 738 kilometers(km) away from Addis Ababa, the capital city of Ethiopia, and 182 km away from Bahir Dar, the capital city of Amhara National Regional State. Gondar university’s comprehensive and specialized hospital is one of the biggest teaching hospitals in the country which serves more than 5 million people living in Northwest Ethiopia. As recorded in the medical database, the hospital gives different services for about 7000 patients per month and more than 84,000 patients per year.

### Source and study populations

All adult patients attending Gondar university’s comprehensive and specialized hospital were the source population and all adult patients aged ≥ 18 years who were presented during the data collection period were the study population. Patients with severe illness and difficulties of communication were excluded from the study.

### Sample size determinations

The sample size for the proportion of willingness to donate cornea was calculated using the single population proportion formula with the following assumptions: margin of error (d) = 5%, confidence level = 95%, non-response rate = 10%, and the proportion of willingness to donate cornea from a similar study as p = 37.60%.

Finally, Total sample size for objective one (n) = (Za/2)^2^(P)(1-P)/d^2^, (1.96)2 (0.376) (0.624)/0.0025 = 398.

The sample size for factors (objective two) was also calculated for the factors that were consistently associated with the willingness of corneal donation in other similar studies. These were “higher educational status” and “having the awareness to donate cornea”. Using Epi info version 7 software and taking a 1:1 ratio between exposed and non-exposed, 80% power, and 95% confidence level, the sample sizes calculated were 234 and 410 respectively for the two factors. After comparing the sample sizes for the two objectives, the largest sample size was 410 and by adding a 10% non-response rate, the final sample size was calculated as 451.

### Sampling technique and procedure

A systemic random sampling technique was used to select study subjects after calculating the interval(K) by dividing the estimated number of patients visiting the hospital by the sample size calculated (7500/451 = 16). By using the lottery method to draw a number from 1–16 that is used to select the first participant, then all patients found by adding the interval 16 were included in the study.

### Operational definitions

Awareness of corneal donation was defined as when the participant had heard of eye donation.

Willingness to donate cornea was defined when the participant had the interest to pledge to donate his/her cornea/eye.

Good Knowledge was defined if the study participants score the mean and above of the 10 knowledge related questions.

Poor knowledge was defined as if the study subjects score below the mean of the 10 knowledge-related questions.

Favorable attitude towards corneal donation was defined as if a study participant scored the mean and above of the 6 attitude-related questions.

An unfavorable attitude towards corneal donation was determined if a study participant scored below the mean of the 6 attitude-related questions.

### Data collection tool and procedures

The data were collected with face-to-face interviews using a pre-tested structured questionnaire which was taken from previous studies. The questionnaire contained information about socio-demographic characteristics of study participants as well as awareness, knowledge, attitude, and willingness about corneal donation. The English version of the questionnaire was translated into Amharic (local language) version for data collection and translated back to English by language experts for consistency. Data were collected by 6 trained optometrists and two supervisors.

### Data processing and analysis

The collected data were entered into Epi info version 7 and exported to statistical package for social science (SPSS) version 20 for formal statistical analysis. Median with interquartile range, frequency, percentage and tables were used to present the summary statistics. The statistical association between dependent and independent variables was analyzed using a binary logistic regression model. In bi-variable logistic regressions variables with a P-value of < 0.20 were entered into a multivariable logistic analysis and those variables with a p-value of p < 0.05 were taken as statistically significant. The fitness of the model was checked with the Hosmer-Lemeshow model fitness test. The odds ratio with 95% CI was used to show the strength of association between independent variables and willingness to donate cornea.

### Ethical considerations

Ethical clearance was obtained from the University of Gondar College of Medicine and Health Sciences, School of Medicine, ethical review committee. After informing about the objective of the study, written informed consent was obtained from each study participant. Confidentiality of the information obtained was assured and maintained through anonymous. Their full right to withdraw or refuse to participate in the study was respected. Finally, the collected data was securely locked.

## Results

### Socio-demographic characteristics

A total of 408 study subjects participated in this study with a response rate of 90%. The median age of the participants was 40 years (range of 19–87). Among the participants, 292 (71.6%) were females and 346 (84.8%) were Christians. More than half, 244 (59.8%) of the participants were married and one-fourth, 102 (25.0%) of them had a monthly average income of less than 1000 Ethiopian Birr (ETB) (Table 1).

**Table 1:** Sociodemographic characteristics of patients attending Gondar University Comprehensive and Specialized Hospital, July 2020.

Variables	frequency	percent
**Age**		
18–30	91	22.3
31–43	145	35.5
> 43	172	42.2
**Sex**		
Male	116	28.4
Female	292	71.4
**Ethnicity**		
Amhara	346	84.8
Non-Amhara	62	15.2
**Religion**		
Christian	346	84.8
Muslim	62	15.2
**Marital status**		
Single	113	27.7
Married	244	59.8
Divorced	8	2.0
Widow	43	10.5
**Education**		
No formal education	116	28.4
Primary school	59	14.5
Secondary school	116	23.0
College university and above	139	34.1
**Occupational level**		
Government employee	131	32.1
Merchant	127	31.1
Farmer	78	19.1
Daily laborer	10	2.5
Housewife	46	11.3
Student	9	2.2
Others	7	1.7
**Monthly Income**		
Below 1000	102	25.0
1000–2000	91	22.3
2000–3000	59	14.5
Above 3000	156	38.2
**Residency**		
Urban	275	67.4
Rural	133	32.6

Others = retired, priest.

### Awareness and knowledge about corneal donation

More than half, 228 (55.90%) of participants had heard about cornel donation. From those participants who had awareness about corneal donation, nearly one-third, 124 (30.4%) of them had good knowledge scores. Only 27 (6.6%) of the participants had gotten information about a corneal donation from multimedia and 82 (20.1%) of the participants had gotten information from health professionals (Table 2).

**Table 2:** Awareness, knowledge and attitude about corneal donation of patients attending Gondar University Comprehensive and Specialized Hospital, July 2020.

Variables	Frequency	Percent
**Awareness**		
Yes	228	55.9
No	180	44.1
**Level of knowledge**		
Poor	104	25.5
Good	124	30.4
**Attitude**		
Favorable	183	44.9
Unfavorable	225	55.1

### Willingness towards corneal donation

Among the study participants, 179 (43.9%), (95% CI: 39.2%–48.5%) respondents were willing to donate their cornea/eye. Most of the study participants (64.5%) were also willing to donate the pledged cornea of their close relatives upon death. Less than one-third of the participants (28.7%) had thought that donating cornea helps other individuals with blind eyes/corneal opacities and 8.6% of the participants believed that donating cornea is the practice of noble work. The most common reasons for unwillingness to donate cornea were requiring more information about corneal donation (17.6%), religious restrictions (17.6%), and requiring to be embedded with an intact body (8.8%) respectively (Table 3).

**Table 3:** Willingness to donate cornea and perceived reasons among patients attending University of Gondar Comprehensive and Specialized Hospital, July 2020.

Variables	Frequency	Percent
**Willingness to donate own cornea**		
Yes	179	43.9
No	229	56.1
**Willingness to donate pledged cornea**		
Yes	263	64.5
No	145	35.5
**Reasons to be willing to donate cornea**		
To help the blind	117	28.7
It is a noble work	35	8.6
Both pleasure and noble work	27	6.6

### Factors associated with willingness to donate cornea

In this study monthly income, attitude towards corneal donation, knowledge of corneal donation, awareness of corneal donation, residency, educational status, marital status, religion, ethnicity, sex, and age were independently associated with the willingness of corneal/eye donation with a p-value of less than 0.20.

After controlling the confounders, variables including age group greater than 43 years, having the religion of Christianity and good knowledge about corneal donation were statistically significant with the willingness of corneal/eye donation. As a result, participants with an age group of greater than 43 years were 68.9% less likely (AOR = 0.311 (95% CI: 0.11–0.89) to donate cornea as compared to the younger ones. Participants who had a religion of Christianity were 3.23 times (AOR = 3.23 (95% CI: 1.09–9.57)) more likely to donate their cornea as compared to those who had a religion of Islam. Besides, those participants who had good knowledge about corneal donation were 5.48 times (AOR = 5.48 (95% CI: 2.69–11.17)) more likely to donate cornea as compared to those who had poor knowledge about corneal donation (Table 4).

**Table 4:** Factors associated with willingness to donate cornea among patients attending University of Gondar Comprehensive and Specialized Hospital, July 2020.

Variables	Willingness to donate cornea		COR 95% CI	AOR 95% CI
	Yes	No		
**Age**				
18–30	48	43	1	1
31–43	77	68	1.04(0.600–1.715)	
>43	54	118	0.410(0.243–0.691)	0.311(0.109–0.887) *
**SEX**				
Male	38	78	1	
Female	141	151	1.917(1.221–3.008)	
**ETHNICITY**				
Amhara	160	186	1.947(1.090–3.476)	
Non-Amhara	19	43	1	
**RELIGION**				
Christian	163	183	2.561(1.396–4.698)	3.229(1.090–9.569) *
Muslim	16	46	1	1
**Marital status**				
Single	76	37	2.591(1.264–5.324)	
Married	81	163	0.628(0.35–1.212)	
Divorced	3	5	0.758(0.160–3.581)	
Widowed	19	24	1	
**educational level**				
No formal education	24	92	0.142(0.080–0.251)	
Primary school	17	42	0.220(0.114–0.427)	
Secondary school	48	46	0.568(0.333–0.969)	
College university &above	90	49	1	
**Residency**				
Urban	135	140	1.950(1.267–3.003)	
Rural	44	89	1	
**Awareness**				
Yes	133	95	4.078(2.664–6.244)	
No	46	134	1	
**History of eye examination**				
Yes	66	40	2.760(1.748–4.357)	
No	113	189	1	
**Knowledge**				
Poor	36	68	1	1
Good	97	27	6.786(3.771–12.211)	5.478(2.685–11.174) *
**Attitude**				
Good	135	90	4.739(3.078%-7.294%)	
Poor	44	139	1	
**Monthly income**				
≤ 1000	35	67	**1**	
1000–2000	21	70	0.574(0.304–1.085)	
2000–3000	26	33	1.508(0.782–2.908)	
> 3000	97	59	3.147(1.869–5.301)	

* = p- value < 0.05.

## Discussion

Unwillingness to donate cornea results in greater demand for corneal transplantation which in turn increases the burden of corneal blindness. The proportion of willingness to donate cornea in this study was 43.9% (95% CI: 39.2–48.1). This finding was found to be higher than other similar studies conducted in Gondar, Ethiopia [20], Singapore [27] and Pakistan [28], The possible explanation might be due to differences in the study setting, i.e. the present study is hospital-based whereas the study done in Gondar was community-based. Furthermore, the majority of the participants in Singapore had poor knowledge about corneal donation when compared to this study and the difference in sampling technique between the above studies might create this variation in proportion. However, this result is lower than similar studies done in Ghana [29], Central Ethiopian [19], and India [18]. This difference might be created due to cultural and socioeconomic differences between the study areas. Also, the awareness level towards corneal donation, the educational level of the participants, the media coverage, and differences in the study settings might contribute a greater impact on the proportion of willingness to donate cornea.

After controlling the effect of confounding, variables in multiple logistic regression, participants who had an age group of greater than 43 years were 68.9% (AOR = 0.311 (95% CI: 0.11–0.89)) less likely to donate cornea as compared to those who were younger than 43 years. This finding is supported by other similar studies done in India [21, 23] which stated that being young was significantly associated with willingness to donate cornea. This might be the fact that older people are strongly attached to cultural and religious beliefs which inhibits them to donate any tissue from their body including the cornea. High willingness in the young’s might probably due to better educational access, better awareness, and relatively higher exposure to mass media. On the other side, older age groups were associated with the willingness of corneal donation in studies done in China [30] and Singapore [27]. This might be explained by differences in age category between the studies. Those who were assumed to be older age groups in the present study were 44 years and above which might be categorized as young in other studies and the study in China has a wider age group. Even though they have the same age classification with this study, age was not at all associated with willingness to donate cornea in studies done in Central and Northwest Ethiopia [19, 20].

The odds of being a willingness to donate cornea among participants who had a religion of Christianity were 3.2 times (AOR = 3.2 (95% CI: 1.09–9.57)) more likely as compared to those participants who had a religion of Islam. This was in agreement with other related studies done in North Kerala, India [31] and Gondar, Northwest Ethiopia (20). On the other side, studies conducted in Singapore [17] and Australia [32] showed that having no religion was significantly associated with the willingness of corneal donation. However, religion was not at all significantly associated with the willingness of corneal donation in studies done in China [30] and Central Ethiopia [19]. The above variations could due to the doctrine/dogma variation between religions, and differences in the practicability of the faith in the followers of the religion.

Participants who had good knowledge about corneal donation were 5.48 times (AOR = 5.48 (95% CI: 2.69–11.17)) more likely to be willing to donate cornea as compared to those participants who had poor knowledge about corneal donation. This was supported by other similar studies done in Saudi [33, 34], Singapore [27], and China [35]. The possible reason could be that knowledge is the precursor of practical activities to be performed. Before promising to donate cornea after death some sort of knowledge about donation is crucial. Therefore, increasing knowledge about corneal donation may be an effective strategy for enhancing the willingness of adults towards corneal donation.

### Limitations

This study was an institution-based study that might overestimate the result. Besides, the study did not include the practices of the participants to give the corneas of their families that had already promised.

## Conclusion

The proportion of willingness to donate cornea was high (43.9%) among participants attending Gondar University Comprehensive and Specialized Hospital. Age group greater than 43 years, a religion of Christianity, and good knowledge about corneal donation were significantly associated with the willingness of corneal donation.

## Supporting information

S1 File Questionnaire and data extraction MS format to the study of willingness of corneal donation and its associated factors among adult patients attending Gondar University Comprehensive and Specialized Hospital. (DOCX)

S2 File (SAV)
