# Supplementary material for: Willingness of corneal donation and its associated factors among adult patients attending Gondar University Comprehensive and Specialized Hospital
Source: PLoS One. 2021 Aug 20;16(8):e0256102. doi: 10.1371/journal.pone.0256102 (PMC8378713; doi:10.1371/journal.pone.0256102)
Supplement: S1 File — (DOCX) [file pone.0256102.s001.docx]

**S1 File:** English and Amharic Version of Questionnaire in a study of Willingness of corneal donation and its associated factors among adult patients attending Gondar University Comprehensive and Specialized Hospital

**Introduction**

Good morning/afternoon, my name is --------------------------------. I am a member of a research group working in Gondar university hospital. I have been studying willingness and associated factors among patients in UOGCSH by using structured questionnaires. Your own answers for all of our questions are important to determine willingness and associated factors towards of the study which is intended. Your answers will be confidential and keep in secret. If you decide that, you do not want to participate in the study now or at any time in the future; it is your right not to participate in the study. But we appreciate if you participate and will take 10-15 minutes for us to complete the questionnaire. Thank you. Next, I will read a consent, which assures your interest to participate.

Do I have your permission to continue?

If yes, thank you and continue --------------

If no, thank you and go to next study subject --------------

Data collector

Name ----------------------- signature ------------------------ date -------------------

Checked by supervisor

Name --------------------- signature---------------------- date-------------------

**Section 1: Participants' socio-demographic characteristics**

|  |  |  |
| --- | --- | --- |
| 1 | Your Age | (years)= |
| 2 | Sex | 1. Male  2. Female |
| 4 | Your religious group | 1.Orthodox  2.Protestant  3.Muslim  4.Catholic  5.Other__________ |
| 5 | Your marital status | 1. Single  2. Married  3. divorced  4. Widowed |
| 6 | Educational level | 1. Can’t read and write  2. Only read and write  3.Primary school  4. Secondary school  5. College and above |
| 7 | Occupation | 1. Government employee  2. merchant  3. Farmer  4. Daily laborer  5. House wife  6. student  7. Others |
| 8 | Family average monthly income | _________________ETB |
| 9 | Residency | 1.urban 2. Rural |
| 10 | Have you had eye examination for the last 2 years? | 1. Yes  2. No |
| 11 | Have you had an eye surgery for the last 2 years? | 1. Yes  2.No |

**Section 2: Participants' awareness about corneal donation**

| 1 | Have you ever heard about corneal donation? | 1. Yes  2. No |
| --- | --- | --- |
| 2 | If your answer is yes in **Q1** What is your source of information? | 1.Health professionals  2. Newspapers/Magazine  3.Television  4. Radio  5.Family member/friends  6.Other ___ |

- If study subjects have no any information about eye donation, please describe what eye donation mean and skip to section 4 (for data collector: Cornea donation is the act of donating the outer transparent part of the eye after one's death for someone to see again) otherwise continue with section 3

**Section 3: Participants' knowledge about corneal donation**

| 1 | If your answer is yes in **Q1**, what do you mean by cornea donation? | 1.Take consent when alive and giving corneas to blind person after one's death  2.Without consent donation of eyes after one's death  3.Donate cornea when alive  4.Do not know  5.Others______ |
| --- | --- | --- |
| 2 | Can cornea be removed from alive person for donation? | 1.Yes  2.No  3.Don't know |
| 3 | Which part of an eye can be removed from the donors during collection? | 1. Whole eye ball  2. Cornea  3. Lens  4. Don't know  5. Others _____________ |
| 4  . | Is it mandatory to get the consent of family members for cornea donation after the death of willing person? | 1. Yes  2. No  3. Don't know |
| 5 | What is the ideal duration to retrieve cornea after the death of a person? | 1. As soon as possible  2. Within 6 hours  3. Within 48 hours  4. Don't know  5. Others ____________ |
| 6 | Can a person who wears spectacles donate cornea? | 1. Yes  2. No  3. Don't know |
| 7 | Can a person who is living with HIV donate his/her own cornea? | 1. Yes  2. No  3. Don't know |
| 8 | 8. every blindness can be treated 1. with corneal donation | 1. Yes  2. No |
| 9 | Is there eye bank in Ethiopia? | 1. Yes  2. No  3. Don't know |
| 10 | Do you think that a human cornea can be bought or sold? | 1. Yes  2. No  3. Don't know |

**Section 4: Participants attitude towards cornea donations and willingness to donate eyes**

| 1 | cornea donation is a pleasure activity to help a blind person? | 1. Strongly disagree  2. Disagree  3. Neither  4. Agree  5.Strongly agree |
| --- | --- | --- |
| 2 | cornea donation is a noble work? | 1.Strongly disagree  2. Disagree  3. Neither  4. Agree  5. Strongly agree |
| 3 | corneal donation is not against religious beliefs? | 1.Strongly disagree  2. Disagree  3. Neither  4. Agree  5. Strongly agree |
| 4 | eye donation cannot delay funeral and religious rites after death? | 1.Strongly disagree 2. Disagree  3.Neither  4.Agree  5.Strongly agree |
| 5 | Corneal donation not cause facial disfigurements? | 1. Strongly disagree  2. Disagree  3. Neither  4. Agree  5. Strongly agree |
| 6 | body is not ill-treated after death due to cornea donation? | 1. Strongly disagree  2. Disagree  3. Neither  4. Agree  5. Strongly agree |
| 7 | Are you willing to donate your cornea? | 1. Yes  2. No  3.I need time to decide  4. I need more information to decide  5. My family may show objection |
| 8 | If your answer is yes, what is your perceived reason to be willing to donate? | 1. It is pleasure to help the blind person  2. Cornea donation is a noble work  3. Cornea donation is both pleasure and noble work  4. My cornea is not useful after death  5. Other__________ |
| 9 | If your answer is No, what is your perceived reason for not to be willing to donate? | 1. I need more information  2. Family members object to cornea donation  3. It is against my religion  4. Feels that body is ill-treated due to cornea donation  5. I want to be body intact after death  6. I have eye problems  7. I am too old  8. Other_____________ |
| 10 | Are you willing to donate close relative's cornea if he/she had pledged to donate? | 1. Yes  2. No  3. I need time to decide  4. I need more information to decide |

## Annex V. Amharic Version of Questionnaires

**የጎንደር ዩኒቨርሲቲ ህክምናና ጤናሳይንስ ኮሌጅ የዓይን ህክምና ክፍል**

መለያቁጥር /ኮድ-----------------

**የመጠየቂያቅፅ**

ጤናይስጥልኝ-------------- እባላለሁ፡፡

የዩነቭርስቲው የጥናት ቡድን አባል ነኝ፡፡የአይን ብልን ልገሳን በተመለከ ተያላቸዉን ፈቃድና ተያያዥ ምክንያቶችን የሆኑ ጎልማሳ ታካሚዎች መካከል ቃለመጠይቅ በማድረግ እያጠናን እንገኛለን፡፡ ይህ ጥናት እርስዎ በሚሰጡን መረጃ ላይ የተመሰረተ ስለሆነ ፍቃድዎ ከሆነ መረጃውን በመስጠት ትብብር እንዲያደርጉልን በትህትና እንጠይቃለን፡፡በጥናቱ ላይ መሳተፍ የማይፈልጉ ከሆነ አሁንም ሆነ በሂደት ውስጥ አለመስማማትይችላሉ፡፡ ሆኖም ግን ጥናቱ ከትንሽ ጊዜ መፍጀት ውጪ ምንም አይነት ጉዳት የማያመጣ ስለሆነ እንዲሳተፉ እናበረታተለን፡፡መረጃዎ ምስጢራዊነቱ የተጠበቀ፣ለጥናቱ ብቻ የሚውልና ለሌላ ጉዳይ የማንጠቀምበት መሆኑን ልናረጋግጥልዎ እንወዳለን፡፡ቃለ-መጠይቁ 15-20 ደቂቃ የሚፈጅ ስለሆነ ፍቃደኝነትዎን በንግግርዎ እንዲያረጋግጡልን በትህትና እየጠየቅን ወደ ቃለመጠይቁ እንሄዳለን፡፡

ማንኛውንም ሊያነሱ የሚፈልጉት ጥያቄ ካለዎት ተመራማሪውን በሚቀጥለው አድራሻ ማነጋገር ይችላሉ፡፡

ስም፡እየሩስገ ሰሰ፣ስ.ቁ፡ 0921403253

**መረጃውን የሰበሰበው**

ስም ----------------------------------- ፊርማ -------------------------- ቀን -------------------

**መረጃውን ያረጋገጠው**

ስም----------------------------------- ፊርማ --------------------------- ቀን ---------------

**ክፍል 1; ስነ ሰብና ማህበራዊ መረጃዎች**

**1**. እድሜዎ በዓመት ______________

**2**. ፆታ 1.ወንድ 2.ሴት

**3.** ብሄርዎ 1.አማራ 2.ትግሪ 3.ቅማንት 4. ሌላ ካለ ይጥቀሱ______________

**4**. ሐይማኖትዎ 1.ኦርቶዶክስ 2.ሙስሊም 3.ፕሮቴስታንት 4.ካቶሊክ 5.ሌላ ______________

**5**. የትዳር ሁኔታ 1.ያላገባ/ች 2.ያገባ/ች 3.የፈታ/ች 4.የሞተችበት/ባት

**6**. የትምህርት ደረጃዎ 1.ማነበብና መፀሐፍ የማይችል 3. 1ኛ ደረጃ ያጠናቀቁ 5.ኮሌጅና ዩኒቨርሲቲ ያጠናቀቁ

2. ማነበብና መፀሐፍ የሚችል 4. 2ኛ ደረጃ ያጠናቀቁ

**7**. ስራዎት 1.የመንግስት ሰራተኛ 2.ነጋዴ 3.አርሶ አደር 4. የቀን ሰራተኛ 5. የቤት እመቤት 6. ተማሪ 7.ሌላ _____

**8**. የቤተሰብዎ ወርሃዊ ገቢ ምን ያህል ነው? በብር _____

**9**. የሚኖሩበት ቦታ? 1.ከተማ 2.ገጠር

**10**. አይኖትን ላለፉት 2ዓመት ውስጥ ታክመው ያውቃሉ? 1.አዎ 2.አይ

**11**. አይኖትን ላለፉት 2ዓመት ውስጥ ቀዶህክምና አድርገው ያውቃሉ? 1.አዎ 2.አይ

**ክፍል 2፡ተሳታፊዎች ስለዓይን ብሌን ልገሳ ያላቸውን ግንዛቤ እና እውቀት ዳሰሳ**

1. ስለ ዓይን ብሌን ልገሳ ከዚህ በፊት ሰምተው ያውቃሉ? 1. አዎ 2. እልሰማሁም

**አልሰማሁም ከሆነ ወደ ክፍል 3**

**2**. ይህንን የመረጃ ምንጭ ከየት አገኙት? 1. ከህክምና ባለሙያ

1. ከሚነበቡ ነገሮች (ከጋዜጣ/ከመፅሄት)
2. ከመገናኛ ብዙሃን (ከቴሌቪዥን፣ከሬድዮ፣ፍስቡክ)
3. ከቤተሰብ/ከጓደኛ
4. ሌላ_____________

**3**. የዓይን ብሌን ልገሳ ማለት ምን ማለት እንደሆነ ያውቃሉ?

1. አንድ ሰው በህይወት እያለ ብሌኑን ለመለገስ በሰጠው የቃል ኑዛዜ መሰረት በሞት በተለየ ጊዜ ብሌኑን ማየት ለተሳነው ሰው መለገስ ማለት ነው፡፡
2. ከዚህ አለም በሞት በተለየ ጊዜ ኑዛዜ ሳያስፈልግ ብሌኑን መለገስ ማለት ነው፡፡
3. በዚህ አለም በህይወት እያለ ብሌኑን ማየት ለተሳነው ሰው መለገስ ማለት ነው፡፡
4. ሌላ _____________________
5. አላውቅም

**4.** አንድ ሰው በህይወት እያለ የዓይን ብሌኑን ማየት ለተሳነው ሰው መለገስ ይችላል?

1. አዎ
2. አይችልም
3. አላውቅም

**5**.የዓይን ብሌኑን መለገስ የፈለገ ሰው የትኛውን የዓይን ክፍል ነው የሚለግሰው?

1. ሙሉ የዓይን ኳሱን
2. ከፊት ጥቁሩን የዓይን ክፍል የሸፈነውን የዓይን መስታወት
3. ከዓይኑ ውስጥ የሚገኘውን ብርሀን አስተላላፊ ክፍል
4. ሌላ _____________
5. አላውቅም

**6.** ሟች ለመለገስ የተናዘዘውን ብሌን ለመውሰድ ከቤተሰቡ ፈቃድ ማግኘት ግዴታ ነው?

1. 1.አዎ
2. 2.ግዴታ አይደለም
3. 3.አላውቅም

**7**.ብሌኑን ለመለገስ የተናዘዘን ሰው ብሌን በምን ያህል ሰዓት ውስጥ መወሰድ አለበት?

1. ወዲያውኑ
2. በ6 ሰዓት ውስጥ
3. በ48 ሰዓት ውስጥ
4. ሌላ
5. አላውቅም

**8**. በመነፅር የሚታከም የዓይን ችግር ያለበት ሰው ብሌኑን መለገስ ይችላል?.

1. አዎ
2. አይችልም
3. አላውቅም

**9**. የኤችአይቪ ቫይረስ በደሙ ያለበት ሰው ብሌኑን መለገስ ይችላል?

1. አዎ
2. አይችልም
3. አላውቅም

**10**.ማንኛውም ዓይነት ዓይነ ስውርነት በብሌን ልገሳ ሊስተካከል ይችላል?

1. አዎ
2. አይችልም
3. አላውቅም

**11**.ኢትዮጵያ ውስጥ አይናቸውን ለመለገስ ፈቃደኛ የሆኑ ሰዎች ብሌን ማስቀመጫ ቦታ(የዓይን ባንክ) አለ?

1. አዎ
2. የለም
3. አላውቅም

**12**.የዓይን ብሌን መሸጥ ወይም መግዛት ይቻላል?

1. አዎ
2. አይችልም
3. አላውቅም

**ክፍል 3፡ተሳታፊዎች ስለ ብሌን ልገሳ ያላቸውን አመለካከትና ለመለገስ ያላቸው ፈቃደኝነት**

|  |  | አልስማማም | በጣም አልስማማም | ገለልተኛ | እስማማለሁ | በጣም እስማማለሁ |
| --- | --- | --- | --- | --- | --- | --- |
| 1 | የዓይንብሌን በመለገስ ማየት የተሳናቸውን ሰዎች መርዳት  አስደሳች ተግባር ነው፡፡ |  |  |  |  |  |
| 2 | የዓይን ብሌን በመለገስ ማየት የተሳናቸውን ሰዎች መርዳት ከሃይማኖት አንፃር ቅዱስ ተግባር ነው፡፡ |  |  |  |  |  |
| 3 | የዓይን ብሌን መለገስ የቀብር ስነስርዓትና ሌሎች ሀይማኖታዊ ተግባራትን አያስተጓጉልም/አያዘገይም፡፡ |  |  |  |  |  |
| 4 | የዓይን ብሌን መለገስ ከሃይማኖት ጋር አይቃረንም፡፡ |  |  |  |  |  |
| 5 | የዓይን ብሌን መለገስ የአስክሬን የፊት ገፅታ አያበላሽም፡፡ |  |  |  |  |  |

7. የዓይን ብሌን ለመለገስ ፈቃደኛ ነዎት?

- 1. አዎ
  2. አይደለሁም

1. ለመወሰን ጊዜ ያስፈልገኛል
2. ተጨማሪ ግንዛቤ ያስፈልገኛል
3. የቤተሰብ ፈቃድ ያስፈልገኛል

8. ለጥያቄቁጥር 7 መልስዎ አዎ ከሆነ ለመለገስ ያነሳሳዎት ምክንያት ምንድን ነው?

1. ማየት የተሳናቸውን ሰዎች መርዳት ስለሚያስደስተኝ
2. ብሌን መለገስ ቅዱስ ተግባር ስለሆነ
3. ብሌን መለገስ አስደሳች ተግባር ስለሆነ
4. ሌላ ___________________

**9**. ለጥያቄ ቁጥር 7 መልስዎ አይደለሁም ለመወሰን ጊዜ ያስፈልገኛል ከሆነ ምክንያት ምንድን ነው?

1.ለመወሰን ተጨማሪ ግንዛቤ ያስፈልገኛል

2.ቤተሰብ ስለ ሚቃወመኝ ነው

3.ሀይማኖቴ ስለ ማይፈቅድልኝ ነው

4.አስክሬኔ ክብር እንዳያጣ ሰግቼ ነው

| 6 | የዓይን ብሌን መለገስ የአስክሬን ክብር አያሳጣም፡፡ |  |  |  |  |  |
| --- | --- | --- | --- | --- | --- | --- |

10. የቤተስብ አባል የሆነ ሰው ቃል የገባዉን ብሌን ለመስጠት ፈቃደኛ ነዎት?

1. አዎ
2. አይደለሁም
3. ለመወሰን ጊዜ ያስፈልገኛል
4. ለመወሰን ጊዜ ተጨማሪ መረጃ ያስፈልገኛል
